# Serum Concentration and Chemotactic Activity of E-selectin (CD62E) in Inflammatory Bowel Disease

**DOI:** 10.1155/S096293519400030X

**Published:** 1994

**Authors:** B. Vainerc, O. H. Nielsen

**Affiliations:** Department of Medical Gastroenterology C Herlev Hospital University of Copenhagen Herlev Ringvej, Herlev Copenhagen DK-2730 Denmark

## Abstract

E-selectin (CD62E) is an endothelial specific glycoprotein belonging to the selectin family of adhesion molecules. Because a high expression of this molecule at intestinal mucosal surfaces in inflammatory bowel disease (IBD) has been described earlier, the aim was to assess serum levels of E-selectin (sE-selectin) and to correlate it to disease activity, and further to evaluate its chemotactic properties at physiological concentrations. Levels of sEselectin were measured by a sandwich ELISA technique in 31 IBD patients together with 15 healthy volunteers. In ulcerative colitis the median value was 0.46 nM (0.16–0.75), in Crohn’s disease 0.47 nM (0.22–1.24), and in healthy controls 0.34 nM (0.22–0.83). No statistically significant differences in sE-selectin were revealed between these groups (p > 0.05). The in vitro chemotactic capabilities of E-selectin (in the concentration range of 0.10–31.4 nM) were assessed using the leading front technique. A significantly increased migratory response was found at concentrations of 1.00 (p < 0.05) and 3.14 nM (p < 0.02). It is concluded that sE-selectin in contrast to sICAM-1 does not act as a sensitive indicator of local immune activation in IBD. However, E-selectin may be important for recruitment and accumulation of neutrophilic granulocytes and other phagocytes involved in the inflammatory process seen in IBD. Future investigations are encouraged in order to reveal its *in vivo* effects.


Mediators of Inflammation 3, 215–218 (1994)

### Serum concentration and chemotactic activity of E-selectin (CD62E) in inflammatory bowel disease

B. Vainer[CA] and O. H. Nielsen

Department of Medical Gastroenterology C, Herlev Hospital, University of Copenhagen, Herlev Ringvej, DK-2730 Herlev, Denmark

[CA] Corresponding Author

**E-SELECTIN (CD62E) is an endothelial specific glycoprotein belonging to the selectin family of adhesion molecules. Because a high expression of this molecule at intestinal mucosal surfaces in inflammatory bowel disease (IBD) has been described earlier, the aim was to assess serum levels of E-selectin (sE-selectin) and to correlate it to disease activity, and further to evaluate its chemotactic properties at physiological concentrations. Levels of sE-selectin were measured by a sandwich ELISA technique in 31 IBD patients together with 15 healthy volunteers. In ulcerative colitis the median value was 0.46 nM (0.16–0.75), in Crohn's disease 0.47 nM (0.22–1.24), and in healthy controls 0.34 nM (0.22–0.83). No statistically significant differences in sE-selectin were revealed between these groups ($p > 0.05$). The *in vitro* chemotactic capabilities of E-selectin (in the concentration range of 0.10–31.4 nM) were assessed using the leading front technique. A significantly increased migratory response was found at concentrations of 1.00 ($p < 0.05$) and 3.14 nM ($p < 0.02$). It is concluded that sE-selectin in contrast to sICAM-1 does not act as a sensitive indicator of local immune activation in IBD. However, E-selectin may be important for recruitment and accumulation of neutrophilic granulocytes and other phagocytes involved in the inflammatory process seen in IBD. Future investigations are encouraged in order to reveal its *in vivo* effects.**

**Key words:** Cell adhesion molecules, Chemotaxis, Crohn's disease, E-selectin, Monoclonal antibodies, Ulcerative colitis, Vascular endothelium

### Introduction

The pathogenesis of the inflammatory bowel diseases (IBD), i.e. ulcerative colitis (UC) and Crohn's disease (CD), is not yet clarified. Among the mediators which in the view of recent research are thought to be of importance for the pathogenesis of these diseases are the adhesion molecules, and among them E-selectin (CD62E) (previously named ELAM-1, endothelial-leucocyte adhesion molecule-1) with a molecular weight of 115 kDa.

The E-selectin molecule is normally not present on the surfaces of vascular endothelial cells, but *in vitro* investigations have revealed that stimulation with certain cytokines (i.e. interleukin-1, tumour necrosis factor-α, lipopolysaccharides, substance P, and interferon-γ) induces production and expression of E-selectin on surfaces of such cells.[1–7] The amount of E-selectin increases shortly after stimulation with these cytokines, whereas it decreases after stimulation for 16–24 h.[4–7] On the contrary, stimulation with TNF-α continuously increases production of E-selectin.[7] After epicutaneous provocation for 2 h *in vivo* with chemicals known to be antigenic in susceptible patients, E-selectin is expressed on the luminal membrane of the vascular endothelial cells. E-selectin amounts to a peak level in approximately 24 h, and then decreases.[8]

E-selectin mediates the binding between endothelial cells, particularly to neutrophilic granulocytes (PMNs),[9] but also to basophilic and eosinophilic granulocytes.[3] It also mediates binding to monocytes[9,10] as well as to a sub-group of T cells ($CLA^+$) and certain strains of tumour cells.[9,11,12] E-selectin has a chemotactic effect, and the activity of E-selectin *in vitro* is crucial for the migration of granulocytes across endothelial monolayers.[9,13]

E-selectin is detectable at the luminal membranes of endothelial cells in active IBD but not in inactive IBD or in healthy volunteers.[14] Accordingly, electron microscopy has shown E-selectin to be exocytosed into the vascular lumen.[15] Therefore, it has been suggested that E-selectin is both a transmembrane protein and a secretory protein.

Antibodies to the E-selectin ligand, $sLe^x$, have been shown to react with venous endothelia of the inflamed appendix,[16] and an analogous mechanism of E-selectin directing leukocytes to inflamed bowel segments may occur in IBD.

The aims of this study was to examine: (1) if E-selectin was present in serum from IBD patients and whether this concentration correlates to disease

© 1994 Rapid Communications of Oxford Ltd activity, and (2) the chemotactic properties of E-selectin on PMNs.

### Patients and methods

*Patients:* Plasma from 31 consecutive patients with well established IBD (16 with UC[17] and 15 with CD[18]), seen in the out-patient clinic, and 15 healthy volunteers, all aged 18–75 years, were studied for E-selectin content using ELISA methods. None had received glucocorticoids within the preceding 4 weeks, and none was colectomized. Patients with known auto-immune diseases or other chronic inflammatory conditions were excluded. Disease activity was scored according to Tvede *et al.*,[19] modified according to Langholz *et al.*[17] and Munkholm *et al.*,[18] by the number of bowel movements per day, the presence of blood, mucus and pus in the stools, abdominal pain, and general symptoms such as malaise, weight loss, fever, and symptoms from joints, skin and eyes (Table 1). Essential clinical parameters are given in Table 2. All patients apart from six (three with UC and three with CD) were treated with 5-aminosalicylic acid-containing drugs (1–4 g/day). Blood from eight healthy volunteers was obtained for purification of PMNs to be used in a chemotactic assay.

*ELISA:* Serum E-selectin (sE-selectin) was analysed by a sandwich enzyme linked immunoassay (ELISA) (R&D Systems Europe Ltd, Abingdon, UK). In brief, two E-selectin monoclonal antibodies, recognizing different epitopes of the E-selectin molecule, were precoated onto polystyrene microtitre wells. Standards or diluted samples were then placed in the wells followed immediately by addition of an enzyme conjugated E-selectin monoclonal antibody. E-selectins in serum or standards were bound to the coating antibody, while the conjugated antibody was bound to another distinct epitope on captured E-selectin. A chromogen solution was added to the wells forming a coloured end product, proportional to the amount of E-selectin in the samples. A conjugate of streptavidin and horseradish peroxidase (Streptavidin–HRP Conjugate, R&D Systems) was used to catalyse this process. The concentration of sE-selectin (nM) was measured by use of a standard curve based on six known concentrations of recombinant E-selectin and an ELISA reader at an absorption of 450 nm (EAR 400, SLT Labinstruments, Salzburg, Austria). Absorption at 630 nm was used for reference. The detection limit was 8.7 pM, and the coefficient of variation 0.5. According to the manufacturer, both monoclonal antibodies were raised specifically against the E-selectin molecule.

**Table 1.** Criteria for determination of inflammatory bowel disease (IBD) activity Symptoms

| Symptoms | IBD activity | | | |
|---|---|---|---|---|
| | Inactive | Mild | Moderate | Severe |
| Bowel movements per day | ≤2 | 3–4 | ≥5 | ≥5 |
| Blood, mucus, pus* | — | < Daily | Daily | Daily |
| Abdominal pain | — | — | Mild | Frequent |
| Extraintestinal symptoms | — | — | Few | Many |

Modified after Tvede *et al.*[19]
* Blood, mucus and pus are not characteristic symptoms in Crohn's disease, regardless of disease activity.

*Isolation of neutrophils:* Peripheral blood was drawn in EDTA (0.2 M). Purification of PMNs was in all cases initiated within 30 min after blood was drawn. After a methylcellulose sedimentation of erythrocytes and haemolysis, leukocytes were washed three times and re-suspended in Gey–albumin solution (2%) at a concentration of $2 \times 10^6$ cells/ml. The final cell suspension contained approximately 80% PMNs, recovery was 70%, and viability after chemotaxis more than 95%.

*Chemotaxis:* E-selectin antigen (200 μg/ml) in PBS (R&D Systems Europe Ltd), Gey's solution, casein (Hammersten, Merck, Darmstadt, Germany), and millipore filters (3 μm pore size, thickness 140 μm in dry condition) (Sartorius, 25N, type SM11302, Göttingen, Germany) were used in the chemotactic assay. E-selectin antigen was diluted with Gey's solution into the following concentrations: 0.10, 0.31, 1.00, 3.14, 10.0, and 31.4 nM. Boyden chambers with cell suspension and E-selectin suspension separated by nitro-cellulose filters were incubated at 37°C for 45 min. Median migratory responses were assessed by the leading front technique, and were based on the analysis of five randomly selected fields as described previously in detail.[20] To estimate spon-

**Table 2.** Clinical parameters of patients with chronic inflammatory bowel diseases

| Subjects | No. | Age* (years) | Sex | Disease activity | | | |
|---|---|---|---|---|---|---|---|
| | | | | Inactive | Mild | Moderate | Severe |
| Healthy controls | 15 | 45 (36–60) | 12F, 3M | — | — | — | — |
| Ulcerative colitis | 16 | 37 (18–75) | 11F, 5M | 5 | 4 | 5 | 2 |
| Crohn's disease | 15 | 42 (22–63) | 9F, 6M | 2 | 2 | 6 | 5 |

*Medians are given with ranges in brackets.

taneous migration (chemokinesis), control experiments were carried out in which PMNs in Gey's solution migrated towards Gey's solution alone (negative control). All results of E-selectin chemotaxis were corrected for chemokinesis. In addition, a positive control was assessed with PMNs in Gey's solution and compared with casein.

*Ethics:* Informed consent was obtained before blood samples were drawn in accordance with the Second Helsinki Declaration. The study was approved by the Scientific Ethical Committee of Copenhagen County.

*Statistics:* Non-parametric statistics (medians and ranges) and Mann–Whitney's rank sum test were applied. A $p$ value less than 0.05 ($2\alpha$) was considered statistically significant.

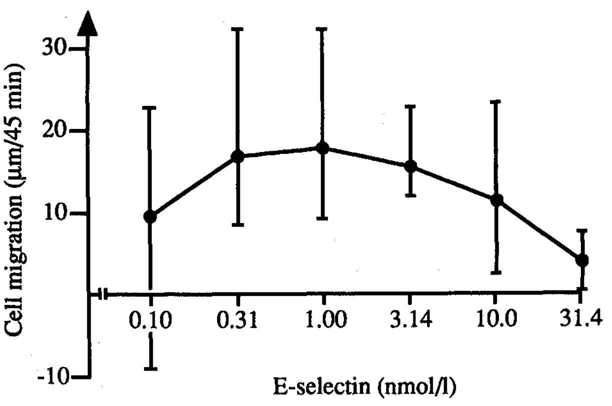

FIG. 2. Chemotactic effect of recombinant E-selectin on PMNs in healthy volunteers given as migration in µm. Medians and 25/75 percentiles are shown. For the concentration 31.4 nmol/l $n = 5$, for the others $n = 8$. The migration ranged from –36.0 to 28.0 µm (at 0.10 nM E-selectin); from –18.0 to 36.5 µm (0.31 nM); from –36.0 to 58.0 µm (1.00 nM); from 7.0 to 28.0 µm (3.14 nM); from –12.0 to 27.0 µm (10.0 nM); and from –24.0 to 37.0 µm (31.4 nM).

### Results

*Serum E-selectin:* The median serum concentration of E-selectin was in UC 0.46 nM (0.16–0.75) ($p > 0.05$) and in CD 0.47 nM (0.22–1.24) ($p > 0.05$) compared with healthy volunteers 0.34 nM (0.22–0.83). Fig. 1 shows sE-selectin concentrations in IBD patients with different disease stages. No correlation to disease activity was revealed ($p > 0.05$). An increase in the concentration of serum E-selectin in IBD patients could not be demonstrated after stratification for sex, age, number of bowel movements per day, presence of blood, mucus and pus in the stools, abdominal pain and presence of extraintestinal symptoms.

*Chemotaxis assays:* The chemotactic effect of E-selectin on peripheral PMNs expressed as the migration in µm/45 min is shown in Fig. 2. Corrected for chemokinesis, the cell migration ranged from –36 to 58 µm in 45 min. Migration had a peak at an E-selectin concentration of 1.00 nM. A significantly increased migratory response corrected for chemokinesis was seen at 1.00 nM ($p < 0.05$) and 3.14 nM ($p < 0.02$, Mann–Whitney's test), whereas at other concentrations no statistically significant differences were detected.

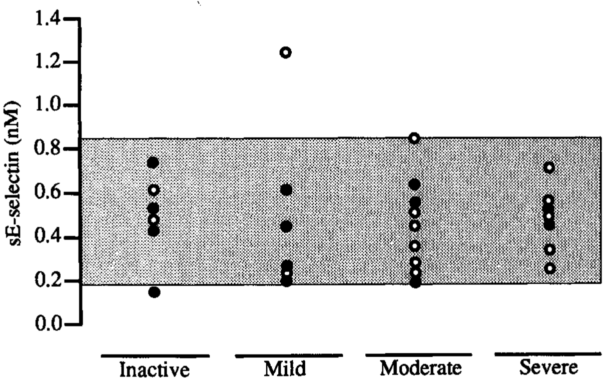

FIG. 1. Concentration of serum E-selectin (nM) among patients with chronic inflammatory bowel diseases, divided on disease activities. The reference range (15 healthy volunteers) is shown (shaded area). Ulcerative colitis (●) and Crohn's disease (○). The median values were 0.46 nM for inactive, 0.35 nM for mild, 0.53 nM for moderate, and 0.49 nM for severe UC; and 0.53 nM for inactive, 0.74 nM for mild, 0.37 nM for moderate, and 0.41 for severe CD. The median value for controls was 0.34 nM.

With regard to the inter-assay variation, a negative value of migration indicated that the spontaneous migration and the E-selectin directed migratory response were identical.

### Discussion

E-selectin has previously been shown to be expressed on the surface of vascular endothelia in a variety of pathological processes located with other tissues, e.g. skin, liver, kidney, gingiva and arteries.[8,21–24] However, none of the included IBD patients had such coexisting diseases.

The results suggest that the sE-selectin concentration is not increased in IBD, and it is therefore likely that sE-selectin in itself is not sufficient to induce and maintain the inflammatory response in IBD. However, it is possible that E-selectin is secreted into the blood stream during acute phases of inflammation and thereby may be involved in the accumulation of PMNs at the inflamed intestinal areas in IBD.

The IBD patients included were allowed to continue 5-aminosalicylic acid treatment. However, the sE-selectin concentrations in six patients not receiving 5-aminosalicylic acid (one with inactive UC, five with moderate or severe IBD) ranged from 19.1 to 70.4 nM. These patients, however, did not influence the overall results. Thus, it is unlikely that 5-aminosalicylic acid may influence E-selectin production.

Paraclinical parameters such as orosomucoid ($\alpha_1$-acid glycoprotein) and haemoglobin are included in the disease activity index described by Tvede *et al.*[19] However, these criteria were not used in our study, as the blood concentration of E-selectin was related only to clinical parameters.

Because a correlation between sE-selectin and disease activity was lacking, sE-selectin cannot be used as an activity marker in IBD. This in contrast to the recent findings that sICAM-1 is significantly increased in active IBD (355 ng/ml), compared with inactive IBD (310 ng/ml) and controls (245 ng/ml) ($p = 0.00002$).[25] Within the different activity stages, a considerable inter-individual variation of E-selectin concentration was found. Further, a large inter-individual variation was seen in healthy volunteers, confirming observations by Fuggle *et al.*[22] in transplanted kidneys.

The chemotactic activity of PMNs in our assay was elicited with physiological levels of E-selectin. The concentrations of sE-selectin were in the range of 0.16 to 1.24 nM, and at these concentrations a chemotactic effect on PMNs was found. The chemotactic effect of recombinant E-selectin has previously been analysed by Lo *et al.*[26] at 10–1 000 nM, showing maximum chemotactic effect at approximately 100 nM. Lo *et al.*[26] did not find any chemotactic effect at concentrations of E-selectin below 10 nM. The present study has revealed such an activity even at concentrations of 0.10 nM, and a significant chemotactic effect was seen at 1 00 nM. Probably, this is due to methodological differences since larger pore size filters (3 μm) were used compared with those used by Lo *et al.*[26], who used 2 μm pore sizes.

From both the present and other results,[24] it is evident that E-selectin by acting with the dual function of transmembrane receptor protein and circulating protein may direct PMNs to inflamed segments in IBD.

Further research on adhesion molecules in IBD patients must be carried out on patients without other known diseases. To avoid a possible influence of anti-inflammatory drugs, investigations should be performed on newly diagnosed IBD patients before initiation of specific anti-inflammatory treatment. Additionally, ***in vivo*** studies of the ability of E-selectin to direct leukocytes across the vascular endothelial cell lining are needed.

### References

1. Kuijpers TW, Hakkert BC, Hoogerwerf M, Leewenberg JFM, Roos D. Role of endothelial leukocyte adhesion molecule-1 and platelet-activating factor in neutrophil adherence to IL-1-prestimulated endothelial cells. *J Immunol* 1991; **147**: 1369–1376.
2. Cronstein BN, Weissmann G. The adhesion molecules of inflammation. *Arth Rheum* 1993; **36**: 147–157.
3. Bochner BS, Luscinskas FW, Gimbrone MA. Adhesion of human basophils, eosinophils, and neutrophils to interleukin 1-activated human vascular endothelial cells: contributions of endothelial cell adhesion molecules. *J Exp Med* 1991; **173**: 1553–1556.
4. Luscinaskas FW, Brock AF, Arnaout MA, Gimbrone MA. Endothelial-leukocyte adhesion molecule-1-dependent and leukocyte (CD11/CD18)-dependent mechanisms contribute to polymorphonuclear leukocyte adhesion to cytokine-activated human vascular endothelium. *J Immunol* 1989; **142**: 2257–2263.
5. Matis WL, Lavker RM, Murphy GF. Substance P induces the expression of an endothelial-leukocyte adhesion molecule by microvascular endothelium. *J Invest Dermatol* 1990; **94**: 492–495.
6. Vidal MJ, Zocchi MR, Poggi A, Pellegatta F, Chierchia SL. Involvement of nitric oxide in tumour cell adhesion to cytokine-activated endothelial cells. *J Card Pharm* 1992; **20**(suppl 12): 155–159.
7. Bevilacqua MP, Pober JS, Mendrick DL, Cotran RS, Gimbrone MA. Identification of an inducible endothelial-leukocyte adhesion molecule. *Proc Natl Acad Sci USA* 1987; **84**: 9238–9242.
8. Friedmann PS, Strickland I, Memon AA, Johnson PM. Early time course of recruitment of immune surveillance in human skin after chemical provocation. *Clin Exp Immunol* 1993; **91**: 351–356.
9. Hakkert BC, Kuijpers TW, Leewenberg JFM, van Mourik JA, Roos D. Neutrophil and monocyte adherence to and migration across monolayers of cytokine-activated endothelial cells: the contribution of CD18, ELAM-1, and VLA-4. *Blood* 1991; **78**: 2721–2726.
10. Leeuwenberg JFM, Jeunhomme TMAA, Buurman WA. Role of ELAM-1 in adhesion of monocytes to activated human endothelial cell. *Scand J Immunol* 1992; **35**: 335–341.
11. Damle NK, Eberhardt C, van der Vieren M. Direct interaction with primed CD4+ CD45R0+ memory T lymphocytes induces expression of endothelial leukocyte adhesion molecule-1 and vascular cell adhesion molecule-1 on the surface of vascular endothelial cell. *Eur J Immunol* 1991; **21**: 2915–2923.
12. Shimizu Y, Newman W, Gopal TV *et al.* Four molecular pathways of T cell adhesion to endothelial cells: roles of LFA-1, VCAM-1, and ELAM-1 and changes in pathway hierarchy under different activation conditions. *J Cell Biol* 1991; **113**: 1203–1212.
13. Carlos T, Kovach, Schwartz B *et al.* Human monocytes bind to two cytokine-induced adhesive ligands on cultured human endothelial cells: endothelial-leukocyte adhesion molecule-1 and vascular cell adhesion molecule-1. *Blood* 1991; **79**: 2266–2271.
14. Koizumi M, King N, Lobb R, Benjamini C, Podolsky DK. Expression of vascular adhesion molecules in inflammatory bowel disease. *Gastroenterology* 1992; **103**: 840–847.
15. Ohtani H, Nakamura S, Watanabe Y, *et al.* Light and electron microscopic immunolocalization of endothelial leucocyte adhesion molecule-1 in inflammatory bowel disease. *Virch Arch A, Pathol Anat* 1992; **420**: 403–409.
16. Munro JM, Lo SK, Corless C, *et al.* Expression of sialyl-Lewis X, an E-selectin ligand, in inflammation, immune processes, and lymphoid tissues. *Am J Pathol* 1992; **141**: 1397–1408.
17. Langholz E, Munkholm P, Nielsen OH, Kreiner S, Binder V. Incidence and prevalence of ulcerative colitis in Copenhagen county from 1962 to 1987. *Scand J Gastroenterol* 1991; **26**: 1247–1256.
18. Munkholm P, Langholz E, Nielsen OH, Kreiner S, Binder V. Incidence and prevalence of Crohn's disease in the county of Copenhagen, 1962–87: a sixfold increase in incidence. *Scand J Gastroenterol* 1992; **27**: 609–614.
19. Tvede M, Bondesen S, Nielsen OH, Rasmussen SN. Serum antibodies to *Bacteroides* species in chronic inflammatory bowel disease. *Scand J Gastroenterol* 1983; **18**: 403–409.
20. Nielsen OH, Elmgreen J. Activation of neutrophil chemotaxis by leukotriene $B_4$ and 5-hydroxyeicosatetraenoic acid in chronic inflammatory bowel disease. *Scand J Clin Lab Invest* 1987; **47**: 605–611.
21. Steinhoff G, Behrend M, Schrader B, Duijvestijn AM, Wonigeit K. Expression patterns of leukocyte adhesion ligand molecules on human liver endothelia. *Am J Pathol* 1993; **142**: 481–488.
22. Fuggle SV, Sanderson JB, Gray DW, Richardson A, Morris PJ. Variation in expression of endothelial adhesion molecules in pretransplant and transplanted kidneys —correlation with intragraft events. *Transplant* 1993; **55**: 117–123.
23. Krugluger W, Lill W, Nell A, Katzensteiner S, Sperr W, Förster O. Lectin binding to chronic inflammatory gingival tissue: possible adhesion mechanisms based on lectin-carbohydrate interactions. *J Periodont Res* 1993; **28**: 145–151.
24. van der Wal AC, Das PK, Tigges AJ, Becker AE. Adhesion molecules on the endothelium and mononuclear cells in human atherosclerotic lesions. *Am J Pathol* 1992; **141**: 1427–1433.
25. Nielsen OH, Langholz E, Hendel J, Brynskov J. Circulating soluble intercellular adhesion molecule-1 (ICAM-1) in active inflammatory bowel disease. *Dig Dis Sci* 1994 (in press).
26. Lo SK, Lee S, Ramos RA, *et al.* Endothelial-leukocyte adhesion molecule 1 stimulates the adhesive activity of leukocyte integrin CR3 (CD11b/CD18, Mac-1, $\alpha_m\beta_2$) on human neutrophils. *J Exp Med* 1991; **173**: 1493–1500.

ACKNOWLEDGEMENTS. The authors are grateful to Richard McGuire, R&D Systems Europe Ltd, for the donation of E-selectin antigen, and to Birgit Dejbjerg and Hanne Kargaard for skilful technical assistance.

**Received 24 February 1994; accepted 14 March 1994**
